# Leopards Exhibit Nuanced Predation Patterns but Rely on Wild Prey in a Human‐Dominated Agricultural Landscape in the Central Highlands of Sri Lanka

**DOI:** 10.1002/ece3.73027

**Published:** 2026-02-11

**Authors:** P. H. Suranga Chanaka Kumara, Andrew M. Kittle, Anjali C. Watson, Sandun J. Perera, Nimalka Sanjeewani, Saminda P. Fernando

**Affiliations:** ^1^ Faculty of Graduate Studies Sabaragamuwa University of Sri Lanka Belihuloya Sri Lanka; ^2^ The Wilderness & Wildlife Conservation Trust Colombo Sri Lanka; ^3^ Department of Natural Resources, Faculty of Applied Sciences Sabaragamuwa University of Sri Lanka Belihuloya Sri Lanka; ^4^ Department of Zoology, Faculty of Natural Sciences The Open University of Sri Lanka Nawala Sri Lanka

**Keywords:** apex predator, conservation, human–leopard interactions, *Panthera pardus kotiya*, prey composition, prey selectivity, Sri Lankan leopard, wild vs. domestic prey

## Abstract

The endemic Sri Lankan leopard is the island's apex predator, living both within and outside protected areas. In unprotected, shared landscapes, it is important to understand leopard diet and predation patterns to foster long‐term human–leopard coexistence. This study examines the diet of leopards in the human‐dominated tea estate landscape of the Upper Kelani River Basin, in Sri Lanka's Central Highlands. Study goals were to evaluate prey composition, diversity, importance and selection, and to investigate the role of domestic species in leopard diet here. Analysis of 107 leopard scat samples showed leopard feeding behavior was best characterized as generalist and opportunistic, with a wide‐ranging diet (*H*′ = 2.89) consisting of 17 evenly consumed (*D* = 0.94) prey species. While the diminutive black‐naped hare (2.5 kg) was most available and most frequently detected in the diet (19.8% of samples), the importance of medium‐sized prey was highlighted, with barking deer (25.5 kg) well utilized (13.9% of samples), representing > 20% of total biomass consumed and showing positive selection (0.281). Moderate selectivity was observed for sambar (0.410), the system's largest potential prey (160–215 kg), which may be expected for meso‐carnivores in the absence of dominant intraguild competition. Primates are a key resource here (23% of samples and biomass) despite being uncommon in tea estates, suggesting preference by leopards. Targeted research to quantify primate abundance and selection is recommended. Overall, wild species represented > 85% of leopard diet, suggesting the landscape retains a substantial natural prey base. Domestic dogs, though common and widely perceived as targeted by leopards here, were moderately avoided (−0.378), a positive outcome for human–leopard coexistence. These findings highlight the leopards' generalist predation tendencies, while suggesting additional complexity and signaling selectivity in predation patterns. Results underscore the necessity of preserving wild prey abundance and diversity to facilitate coexistence in anthropogenically transformed environments.

## Graphical abstract

The Sri Lankan endemic subspecies of leopard ( *Panthera pardus kotiya* ) is facing intensifying human–leopard interactions in unprotected highlands, from where 107 scat samples indicated its wide‐ranging diet of 17 prey species, the black‐naped hare ( *Lepus nigricollis* ) being the favorite, while medium‐sized prey such as barking deer (*Muntiacus malabaricus*) showed a significant prey biomass contribution, whereas domestic species comprised < 15% of the diet.

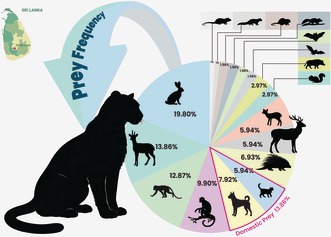

## 1 Introduction

Habitat loss and the reduction of forest cover have had significant consequences globally on wildlife—especially large carnivores—leading to population declines and extirpation which can have cascading effects across trophic levels (Ripple et al. 2014). These impacts typically also lead to increased human–carnivore interactions, with predators increasingly utilizing human‐dominated landscapes in their search for resources (Soni and Selwyn 2023). In Sri Lanka, the widespread loss of endangered large fauna, attributed mostly to habitat degradation and poaching, has long been understood (Amerasinghe and Ekanayake 1992), however, these anthropogenic threats continue at an alarming rate (Gunatilleke et al. 2008; Perera and Fernando 2024).

The endemic Sri Lankan leopard ( *Panthera pardus kotiya* ), the island's largest terrestrial carnivore and apex predator (Kittle et al. 2018), is listed as Vulnerable by the IUCN's Global Red List due to habitat loss/fragmentation and ongoing persecution (Kittle and Watson 2020; Kittle et al. 2021). Leopard habitat suitability in Sri Lanka is closely linked to forest cover and composition, as well as the proximity of protected areas (PAs) which put stringent limitations on human activity (e.g., National Parks and Strict Nature Reserves); however, leopards here also reside in unprotected areas throughout much of the island (Kittle et al. 2018). Given that Sri Lanka is one of the most densely populated countries in Asia (358/km^2^; World Bank Group 2024) and with a leopard population estimated at ~800 adult individuals living within and outside PAs (Kittle and Watson 2020), it is essential that human–leopard coexistence be maintained to ensure the leopard's long‐term viability on the island.

Key to constructing effective carnivore coexistence strategies is understanding predation patterns. Leopards, like other large felids, primarily prey on wild ungulates (Hayward et al. 2006; Baral et al. 2024); however, when wild prey sources are reduced, they are adept at switching predation to domestic species (Karanth and Sunquist 1995; Khorozyan et al. 2015; Athreya et al. 2016). In Sri Lanka, the food preferences of leopards within protected areas have received some research attention (Amerasinghe and Ekanayake 1992; Ranawana et al. 1998; Kittle, Anderson, et al. 2017; Kittle, Watson, and Fernando 2017); however, the diet of leopards outside PAs remains largely unexamined (but see Kittle et al. 2014). Understanding the leopard's diet in regions where they overlap with humans is therefore of high priority, for if leopards are found to be relying on domestic prey species (e.g., goats, cattle, dogs) here, this may intensify negative human–leopard interactions and provide a challenge to lasting coexistence (Athreya et al. 2016; Uduman et al. 2022). Analysis of the leopard's diet in these regions can therefore provide credible insights as to the relative importance of domestic vs. wild prey for leopards and help to guide policy that aims to foster human–carnivore coexistence in shared landscapes.

**FIGURE 1:**
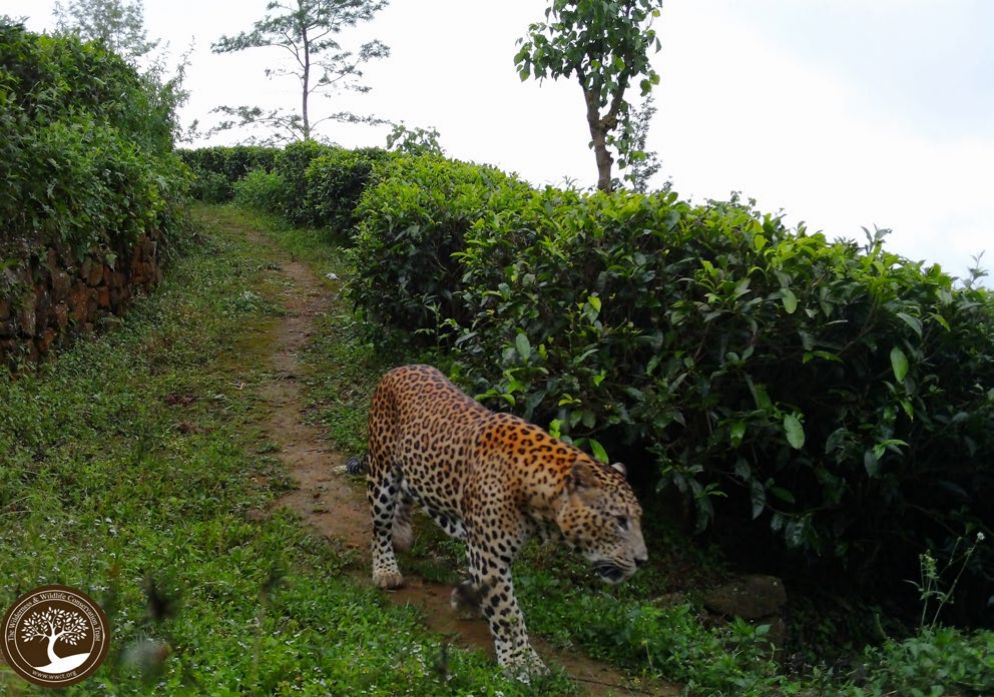
Resident male leopard in Sri Lanka's Central Highlands wanders along an upland path amidst the tea bushes in the early morning. Photo Credit: The Wilderness & Wildlife Conservation Trust.

One such key shared landscape in Sri Lanka is represented by the vast tea plantation lands of the Central Highlands (Kittle et al. 2012, Webb et al. 2020; Figure 1). Here, in a region with a particularly high human population density (486/km^2^, Parliament of Sri Lanka 2021) and relatively few protected areas, human–leopard interactions have intensified (Kumara et al. 2020). This accelerating rate of adverse interactions has the potential to negatively impact the Sri Lankan leopard population in the long term (Kittle et al. 2021).

The current study aims to address the research gap that exists outside PAs by investigating the diet of leopards in an unprotected tea estate landscape in Sri Lanka's Central Highlands. Our overarching goals were to understand leopard diet composition as well as predation and selection patterns here, with an emphasis on the use of wild vs. domestic prey. Specifically, we had two main objectives, with the first to test three alternate hypotheses related to leopard predation patterns and diet: (1) Leopards are generalists and their predation patterns are opportunistic, in which case leopard diet will predominantly consist of those prey species that are most available on the landscape, with no signs of preference; (2) leopards bias predation towards medium‐sized prey (10–40 kg) in keeping with observed global preferences (Hayward et al. 2006), in which case we would expect prey of that size to form the bulk of the diet and/or be preferentially selected; and (3) leopards prefer larger prey in a system devoid of dominant intraguild predators (Kittle, Watson, and Fernando 2017), in which case leopard diet will be biased towards larger available prey species. Our second objective was regarding the role of domestic vs. wild prey species in leopard diet in this human‐dominated landscape, with our hypothesis that wild prey availability is sufficient in the unprotected tea estate landscapes. This would be supported by observing a diet dominated by and/or selection for wild prey species, whereas if wild prey is depauperate in this unprotected landscape, we should see a diet dominated by and/or selection for domestic species. These analyses will help to clarify the ecological role of leopards in landscapes with dense human population and to suggest conservation strategies aimed at reducing negative human–leopard interactions.

## 2 Study Area

**FIGURE 2:**
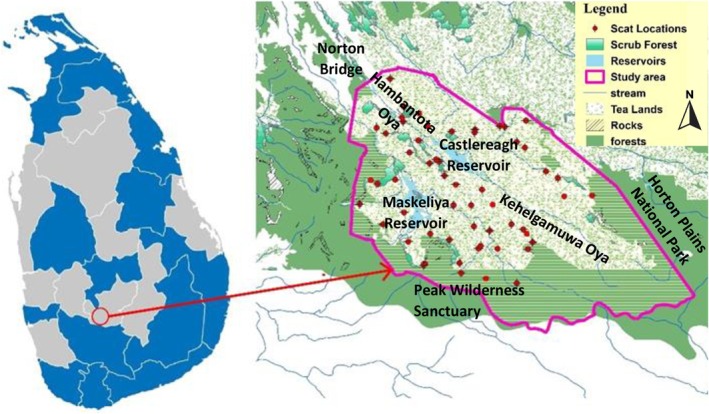
Map of the study area location within Sri Lanka also showing its Districts (left panel) and a detailed map of the study area boundary, highlighting tea lands, rocks, reservoirs, waterways, forests, and scat collection locations (right panel). Scat collection was opportunistic while traversing the landscape during remote camera checks.

The study was conducted within the Ambagamuwa(korale) Divisional Secretariat Division, situated in the western part of the Nuwara Eliya district in the Central Highlands of Sri Lanka, a mountainous region that rises to > 2500 masl (Figure 2). This region is dominated by vast tea plantations, although with a human population density of 528/km^2^ (Ambagamuwakorale Divisional Secretariat 2020). In addition to cultivated tea lands, this diverse environment includes partially degraded secondary forests, grass‐ and shrub‐land where tea cultivation has been abandoned, small plantations of introduced *Pinus* and *Eucalyptus*, pockets of relatively intact natural forest, and human settlements. The study region specifically comprises the upper sections of the Kelani River basin (hereafter, Upper Kelani River Basin; UKRB), focusing on the watershed of Hambantota Oya situated upstream of Norton Bridge, as well as its upstream tributary, Kehelgamu Oya (Figure 2).

## 3 Methodology

### 3.1 Scat Collection

**FIGURE 3:**
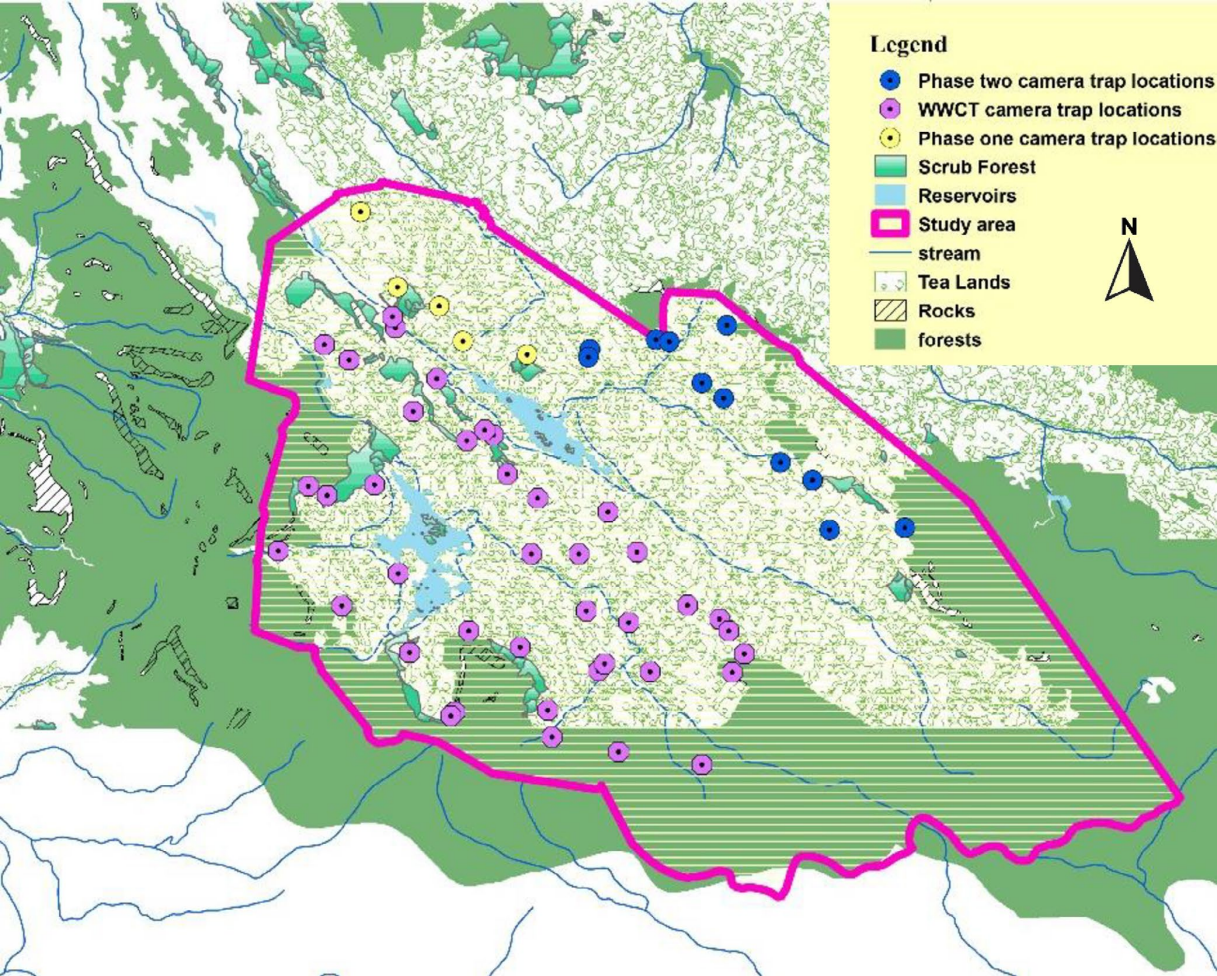
The study area with Phase One camera trap locations from August to October 2021 in yellow, Phase Two camera trap locations from December 2021 to August 2022 in blue, and WWCT camera trap locations from August to December 2016 in purple.

A total of 83 leopard scats were opportunistically collected within the study area between 2017 and 2023 during the course of ongoing remote camera surveys (Figure 3). The remote camera survey design ensured that the entire study area was covered (Karanth and Nichols 1998) with regular visits to check remote cameras further ensuring adequate searching intensity. Remote cameras were set along unpaved tea roads, walking paths and animal trails (Karanth and Nichols 1998), and it was along these that most scat samples were detected. Leopard scats were recognized based on their distinctive “segmented” appearance (Norton et al. 1986), hair content and size, having a bolus width larger than 2.5 cm (Henschel and Ray 2003). This was to rule out the chance that the scats originated from a fishing cat ( *Prionailurus viverrinus* ) or another wild carnivore, but since the leopard is the only large carnivore in Sri Lanka, the chances of mistaking leopard scat for another species here are low (Kittle et al. 2014; Kittle, Watson, and Fernando 2017).

Entire scats were collected, with each sample put into a separate plastic zip‐locked bag and marked in permanent marker with the date, GPS location, description of the site, and names of those who collected it. If scats were fresh or rain had rendered them wet, they were sun‐dried and returned to their designated zip‐locked bag. Sample bags were stored in a Styrofoam cooler box until analysis.

### 3.2 Scat Analysis

Scat samples were initially bathed in warm water to loosen them for cleaning, after which bones, quills, nails, and hooves were identified and manually separated. The remaining samples were then sieved to remove undesirable particles, and the separated hair moved into a clean petri dish according to its unique identifying number, where it was placed in the oven at 60°C for 24 h. Once completely dry, the hair was placed in a new plastic zip‐locked bag and labeled. Separated bones were cleaned, placed in a large Petri dish, and allowed to dry in the sun. Once all were dry, they too were bagged in a fresh plastic zip‐locked bag and labeled to match the hair from the same sample.

Twenty hairs were randomly selected from each sample for analysis (Mukherjee et al. 1994; Sankar and Johnsingh 2002; Farhadinia et al. 2014; Ramesh et al. 2009). The initial analysis aimed at differentiating types of hair on the basis of dimensions, form, and color, and was undertaken in water under a light microscope (Sooriyabandara 2015). Subsequently, a more complete microscopic investigation of cuticular scalation (through scale castings), medulla, and coloration (via temporary water mounts) was done, following methodologies comprehensively outlined in Kittle et al. (2014). If scats had fewer than 20 hairs, all of the hairs were examined. Hairs that could not be identified were classified as unknown, while species to which identified hairs belong were assigned as identified prey species for each scat sample. Bones and other parts (e.g., nails, quills, hoofs) were matched with the identified hair for verification purposes, with bones identified where necessary based on a reference collection housed at the Open University of Sri Lanka.

Data from an additional 23 scat samples previously collected within the study area and analyzed using similar methods (see Situnayake 2018 for procedural details) were added to the 83 samples to get a more complete overview of leopard diet here. Therefore, a total of 107 scats from the study area were analyzed, of which six did not contain hair or identifiable bone parts and hence were classified as unknown. Of the 101 scat samples for which prey species could be determined, 100 samples were identified primarily by hairs, while one sample of Situnayake (2018) was determined by identifying only bones.

Hairs were identified at the genus or species level by applying keys and descriptions provided by Amerasinghe (1983), and comparisons were done with reference hair samples carefully stored at the Wilderness and Wildlife Conservation Trust in Colombo, Sri Lanka (WWCT). The common names of identified species adhered to conventions specified in Kotagama and Goonatilake (2013). Furthermore, for the assessment of the conservation status of each species, reference was made to Weerakoon (2012), while biogeographic status data were gathered from Dittus (2017).

### 3.3 Prey Diversity and Biomass

The Shannon–Wiener Diversity Index (*H*′), which is used to estimate the average diversity in a large collection based on the proportions of species (Magurran 2004), was used to assess species diversity in the leopard's diet:

$$H^{\prime }=-\sum \left({p_{i}}^{*}\;\ln\;\left(p_{i}\right)\right)$$

where *p*_*i*_ is the proportion of each prey species found in the scat samples. The relative frequencies of each species were used to calculate *p*_*i*_. A low *H*′ value, nearer to 0 specifies low diversity and a high *H*′ value, approaching 4 or higher, shows high diversity.

The Simpson's Diversity Index (*D*) (Simpson 1949) was calculated to measure species dominance within the leopard's diet:

$$D=\sum \left({p_{i}}^{2}\right)$$

where *p*_*i*_ is the proportion of each prey species. Ranging from 0 to 1, a higher *D* value specifies lower diversity (i.e., higher dominance by few species).

To get a more comprehensive understanding of the importance of prey species within a leopard's diet it is valuable to estimate biomass consumption in addition to frequency. The regularity of prey species' appearance in a leopard's diet is determined using prey frequency, whereas the total weight of the consumed prey species can be estimated by calculating the prey biomass. This can show, for example, that consuming one large prey species (e.g., sambar) provides more energy and nutrition—and is therefore more valuable to the leopard (Sooriyabandara 2015) than consuming multiple smaller prey species (e.g., rodents).

Traditional prey biomass models have been linear (e.g., Ackerman et al. 1984), but these have been shown to be biased for tropical large carnivores as they underestimate the consumption of medium‐sized prey (Chakrabarti et al. 2016). More recently, a leopard‐specific, nonlinear model has been introduced to better reflect limits related to food intake and scat production (Lumetsberger et al. 2017). This model applies a Michaelis–Menten function to determine a correction factor (CF) which represents the saturation effect in prey consumption where the biomass consumed per scat reaches an asymptote regardless of increasing prey body mass. This model is more widely used in leopard diet studies (e.g., Kandel et al. 2020; Havmøller et al. 2021) and was the model utilized here. It is expressed as:

$$\mathrm{CF}1\text{leopard}=\left(2.242\times x\right)/\left(4.976+x\right)$$

where *x* is the prey body mass. The model illustrates that there is an ideal efficiency in food consumption that does not increase proportionally with additional prey mass.

### 3.4 Prey Availability and Preference

Jacob's Index (Jacobs 1974) was used to determine prey preference or avoidance:

$$D=\left(r-p\right)/\left(r+p-2\mathrm{rp}\right)$$

where *r* is the proportion of a prey species in the leopard's diet and *p* is the proportion of that prey species available in the environment. The Jacob's index values range from +1 to −1 with +1 representing strong preference and −1 complete avoidance. Lack of selection or avoidance (i.e., prey included in diet in proportion to availability) is represented by values near 0 (Jacobs 1974).

To determine prey availability, an array of camera traps was used, with cameras set at 56 locations across the study area (Figure 3). In the 2021–2022 period, 16 locations were utilized across 2 phases to cover the section of the study area north of the Hambantota Oya and Kehelgamu Oya which respectively flow into and out of the Castlereagh reservoir. We also utilized previous camera trap data from 2016 from 40 remote camera locations in the southern part of the study area—south of the Hambantota Oya and Kehelgamu Oya (Kittle and Watson, unpublished data, see Appendix S1 for camera trap locations; Figure 3).

Cameras were set beside tea roads, walking paths and animal trails, with the lens focused across the tracks to capture all passing animals. As animals moved through the trap detection range, photos were captured. Most camera stations had two cameras, one on each side of the trail, for although here were used to estimate prey availability, they were concurrently being used to identify individual leopards. Camera traps were active 24‐h a day (with each 24‐h period denoted as a “trap night”). Animals captured after a 10 min interval (Tanwar et al. 2021) from the previous camera trap photocapture of the same species were considered different individuals.

For each species the relative abundance index (RAI) was calculated:

$$\mathrm{RAI}=\left(\text{Number of captures of}\;a\;\text{species}/\text{Total camera trap nights}\right)\times 100$$

The RAI provides a measure of the relative abundance of prey species in the UKRB, which is essential for assessing the availability of prey species.

It is important to note that since camera traps are set to detect medium‐to‐large terrestrial species, it is not possible to determine the relative abundance of arboreal species (e.g., primates) or very small species (e.g., rodents). As such, for prey availability analyses, only those species that are commonly detected by camera traps (i.e., medium‐to‐large terrestrial species) were utilized.

## 4 Results

### 4.1 Species Composition and Frequency in the Leopard's Diet

Prey species in only six leopard scat samples (5.6% of the total of 107 scats) could not be identified due to the absence of hair and unrecognizable fragmented bone parts. From the 101 scat samples, which contained prey hair or identifiable bones, 107 distinctive prey items were extracted. Six scats were mixed samples containing items from two prey species. Of these, porcupine ( *Hystrix indica* ) was found in four mixed samples, with domestic dog ( *Canis lupus familiaris* ), wild boar ( *Sus scrofa* ), Sri Lanka toque monkey ( *Macaca sinica* ), and Sri Lanka white‐spotted mouse deer ( *Moschiola meminna* ) respectively. The two remaining mixed samples both contained black‐naped hare ( *Lepus nigricollis* ) and domestic cat ( *Felis catus* ).

**FIGURE 4:**
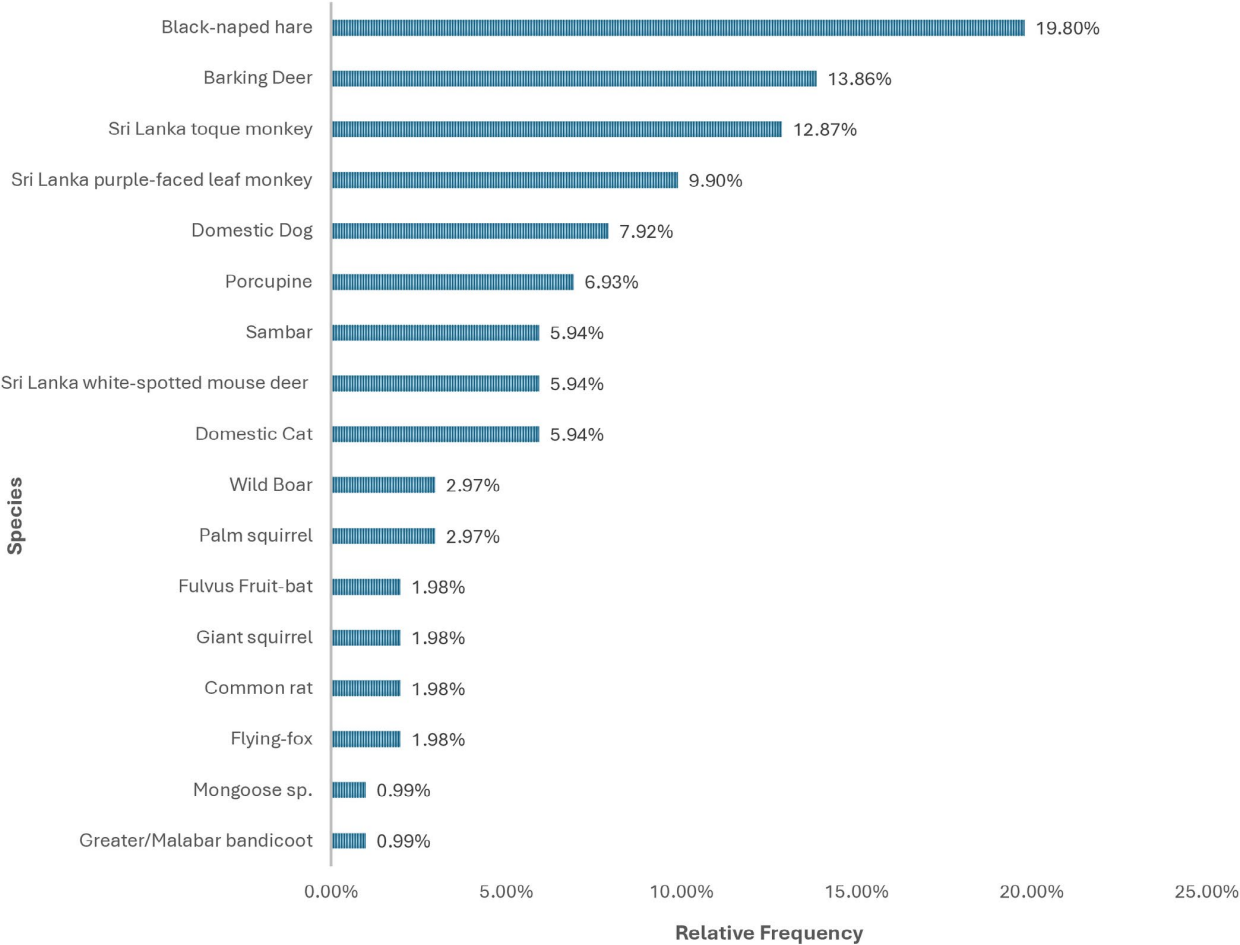
Frequency of prey species found in leopard diets (*N* = 101 scat samples).

A total of 17 prey species were identified (Table 1; Figure 4). However, for certain closely related species (i.e., mongoose, *Urva* sp.), separation at the species level was not possible, so the total species number may be higher. This diverse collection encompasses 6 Orders: Rodentia (5 species), Artiodactyla (4 species), Carnivora (3 species), Primata (2 species), Chiroptera (2 species), and Lagomorpha (1 species). Notably, most of these species are characteristic of forest habitats, with domestic dogs and cats associated with residential situations. Many of the Rodentia as well as the *Urva* sp. are associated with both areas.

**TABLE 1:** Details on identified prey species from leopard scat, and their relative frequency in scat samples.

	Prey species	Order	Conservation status^a^	Biogeographic status^b^	Relative frequency (%)
1	*Bandicota indica indica* Greater/Malabar bandicoot	Rodentia	Least Concern	Native	0.99
2	*Canis lupus familiaris* Domestic dog	Carnivora	Least Concern	Introduced	7.92
3	*Felis catus* Domestic cat	Carnivora	Least Concern	Introduced	5.94
4	*Funambulus palmarum* Palm squirrel	Rodentia	Least Concern	Endemic	2.97
5	*Urva* sp. Mongoose species	Carnivora	Least Concern	Endemic	0.99
6	*Hystrix indica* Porcupine	Rodentia	Least Concern	Native	6.93
7	*Lepus nigricollis* Black‐naped hare	Lagomorpha	Least Concern	Endemic	19.80
8	*Macaca sinica* Sri Lanka toque monkey	Primates	Critically Endangered	Endemic	12.87
9	*Moschiola meminna* Sri Lanka white‐spotted mouse deer	Artiodactyla	Least Concern	Endemic	5.94
10	*Muntiacus malabaricus* Barking deer	Artiodactyla	Near Threatened	Native	13.86
11	*Pteropus medius* Flying fox	Chiroptera	Least Concern	Native	1.98
12	*Rattus rattus* Common rat	Rodentia	Least Concern	Endemic	1.98
13	*Ratufa macroura* Giant squirrel	Rodentia	Least Concern	Endemic	1.98
14	*Rousettus leschenaulty* Fulvus fruit bat	Chiroptera	Least Concern	Native	1.98
15	*Rusa unicolor* Sambar	Artiodactyla	Near Threatened	Endemic	5.94
16	*Semnopithecus vetulus* –Sri Lanka purple‐faced leaf monkey	Primates	Critically Endangered	Endemic	9.90
17	*Sus scrofa* Wild boar	Artiodactyla	Least Concern	Native	2.97

^a^ Weerakoon (2012).

^b^ Dittus (2017).

The prey species most frequently identified in leopard scat was black‐naped hare, followed by barking deer (*Muntiacus malabaricus*) and Sri Lanka toque monkey. Domestic species were found in < 15% of samples. Sri Lanka purple‐faced leaf monkey ( *Semnopithecus vetulus* ), porcupine, Sri Lanka white‐spotted mouse deer, and sambar ( *Rusa unicolor* ) were the other species with > 5% representation (Figure 4).

### 4.2 Prey Diversity and Dominance

The Shannon–Wiener Diversity Index (H′) value of 2.89 shows that the leopard's diet in this landscape is relatively diverse, with many prey species contributing. The Simpson's Diversity Index (D) value (0.94) reflects the absence of any single species dominating the diet, suggesting a balanced representation among these prey species.

### 4.3 Prey Biomass

**FIGURE 5:**
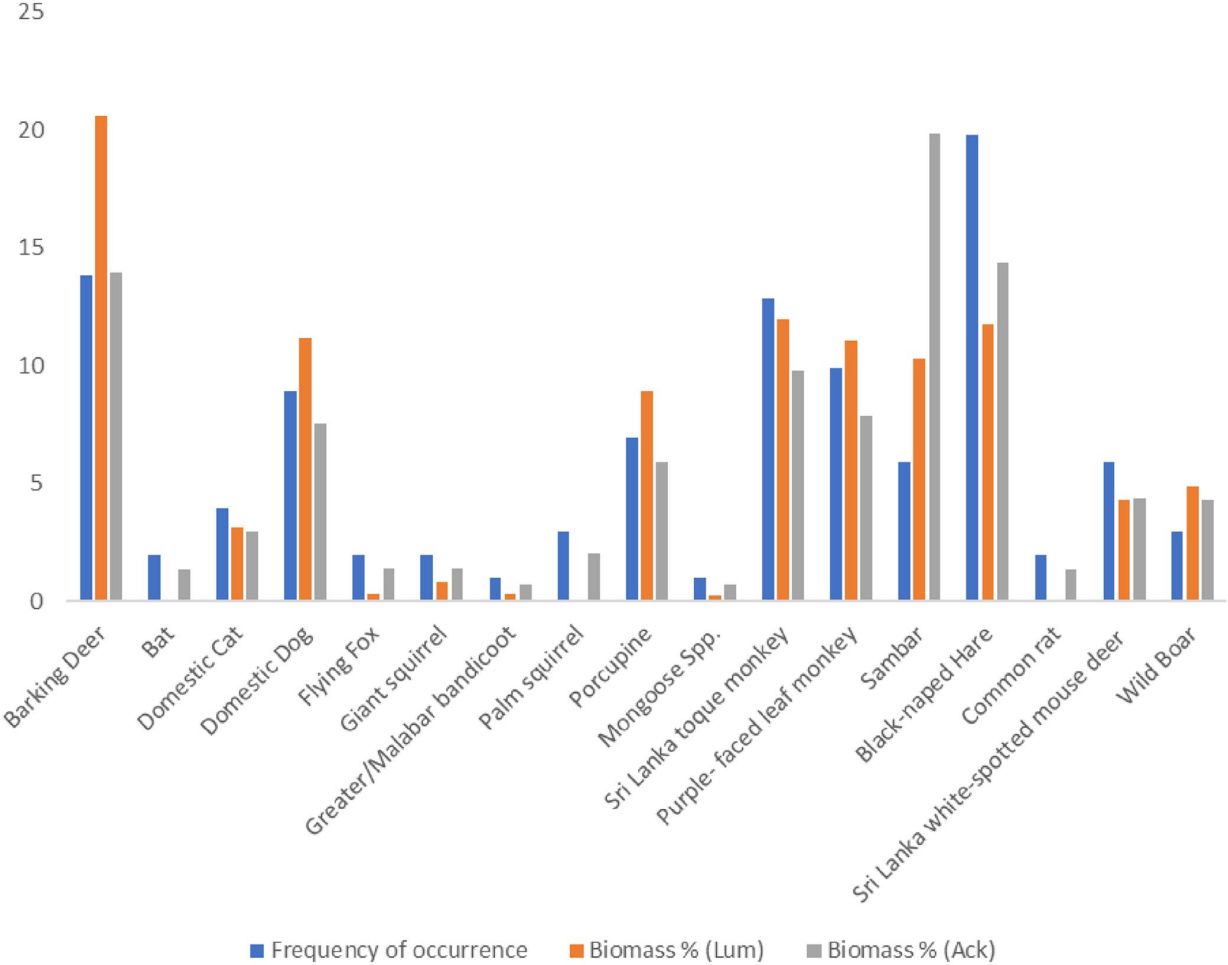
Contribution of consumed prey species to leopard diet based on biomass, calculated using frequency of occurrence and average prey weight in the Upper Kelani River Basin, Sri Lanka.

Barking deer showed the highest contribution of biomass to leopard diet (20.6%), with six other species—Sri Lanka toque monkey, black‐naped hare, domestic dog, Sri Lanka purple‐faced leaf monkey, sambar and porcupine—contributing ~9%–12% of biomass (Table 2; Figure 5; see Appendix S2 for model validation including the Figure S1, residual plot and Figure S2, model and Appendix S3 for the relationship of prey weight with biomass contribution and capture frequency, where Figure S3 regresses prey weight vs. biomass contribution and Figure S4 plots prey weight vs. frequency of prey capture).

**TABLE 2:** Biomass contribution of prey species based on frequency and average weight, assessed using Lumetsberger et al.'s (2017) non‐linear equations.

Species	Average weight	Source	Frequency of occurrence (FoO)	Correction factor (CF) (Lumetsberger et al. 2017)	FoO × CF (Lumetsberger et al. 2017)	Biomass % (Lumetsberger et al. 2017)	Correction factor (Ackerman et al. 1984)	FoO × CF (Ackerman et al. 1984)	Biomass % (Ackerman et al. 1984)
Barking deer	25.5	Kittle et al. (2021)	13.86	1.8759	26.0005	20.6039	2.8725	39.8129	13.9790
Fulvus fruit bat	0.02	Altringham (1996)	1.98	0.0090	0.0178	0.0141	1.9807	3.9218	1.3770
Domestic Cat	4	McCune (2010), Liberg (1982)	3.96	0.9991	3.9565	3.1353	2.12	8.3952	2.9477
Domestic dog	12	Wang and Macdonald (2009)	8.91	1.5848	14.1208	11.1899	2.4	21.3840	7.5083
Flying fox	0.44	Hossain et al. (2013)	1.98	0.1821	0.3606	0.2858	1.9954	3.9509	1.3872
Giant squirrel	1.5	Sooriyabandara (2015)	1.98	0.5193	1.0282	0.8148	2.0325	4.0244	1.4130
Greater/Malabar bandicoot	0.97	Sooriyabandara (2015)	0.99	0.3657	0.3621	0.2869	2.01395	1.9938	0.7001
Palm squirrel	0.07	Sooriyabandara (2015)	2.97	0.0311	0.0924	0.0732	1.98245	5.8879	2.0673
Porcupine	13	Kittle et al. (2021)	6.93	1.6214	11.2362	8.9040	2.435	16.8746	5.9249
Mongoose species	0.93	Hussain and Mahmood (2016)	0.99	0.3530	0.3495	0.2770	2.01255	1.9924	0.6996
Sri Lanka toque monkey	5.5	Dittus (2017)	12.87	1.1771	15.1489	12.0046	2.1725	27.9601	9.8173
Sri Lanka purple‐faced leaf monkey	8.4	Sooriyabandara (2015)	9.9	1.4080	13.9388	11.0457	2.274	22.5126	7.9046
Sambar	215	Kittle et al. (2021)	5.94	2.1913	13.0162	10.3146	9.505	56.4597	19.8240
Black‐naped Hare	2.5	Kittle et al. (2021)	19.8	0.7497	14.8447	11.7636	2.0675	40.9365	14.3735
Common Rat	0.12	Sooriyabandara (2015)	1.98	0.0528	0.1045	0.0828	1.9842	3.9287	1.3794
Sri Lanka white‐spotted mouse deer	3.45	Sooriyabandara (2015)	5.94	0.9180	5.4528	4.3210	2.10075	12.4785	4.3814
Wild boar	61.67	Kittle et al. (2021)	2.97	2.0746	6.1616	4.8827	4.13845	12.2912	4.3157

### 4.4 Prey Preference

With all the camera trap sessions, the total number of trap nights was 3394. Black‐naped hare had the highest RAI (23.6), followed by domestic dog (18.4), porcupine (12.5), and barking deer (10.2). Of the potential prey species monitored with camera traps, domestic cats were the rarest (RAI = 0.3), with sambar also relatively uncommon (RAI = 3.2; Table 3).

**TABLE 3:** Captured prey species from camera traps and their Relative Abundance Indices (RAIs).

Species common name	No of captured species	Proportion	Frequency	Relative abundance Index	Relative frequencies	Jacobs index
Barking Deer	345	0.123	14	10.164	0.200	0.281
Domestic Cat	11	0.004	6	0.324	0.086	0.918
Domestic Dog	623	0.222	8	18.355	0.114	−0.378
Porcupine	425	0.152	7	12.522	0.100	−0.235
Sambar	107	0.038	6	3.153	0.086	0.410
Black‐naped Hare	800	0.286	20	23.571	0.286	0
Sri Lanka white‐spotted mouse deer	210	0.075	6	6.187	0.086	0.074
Wild Boar	281	0.100	3	8.279	0.043	−0.424

**FIGURE 6:**
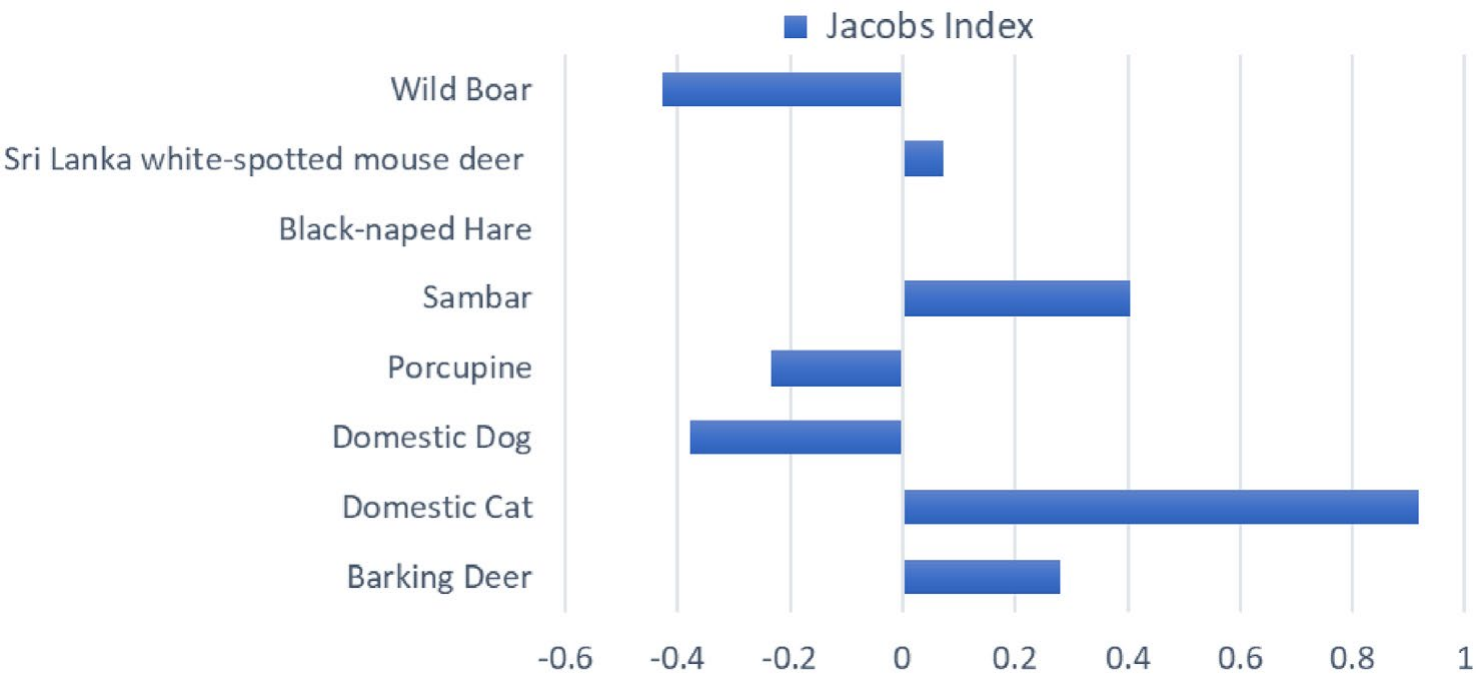
Bar plot showing the Jacob's Index of prey species preference. This was determined by considering prey species identified in scats (*N* = 101), with regard to their relative abundance in the area (species captured in camera traps, *N* = 3394 trap nights). Values close to −1 indicate avoidance and closer to 1 show preference, while values around zero indicate neutral and are consumed relative to their availability. Extreme values occurred when prey species occurred in the scats but were not seen in the cameras (+1) or when there were species seen in the cameras that did not occur in the scats (−1).

Jacob's Index results (Figure 6) showed that the domestic cat was the prey species with the highest preference index (0.918). Sambar showed a moderate level of selection (0.410), with barking deer also having a positive index value (0.281), whereas wild boar (−0.424), domestic dogs (−0.378), and porcupines (−0.235) showed moderate levels of avoidance. Black‐naped hare (0.000) and Sri Lanka white‐spotted mouse deer (0.074) were neither avoided nor preferred but predated in accordance with availability.

## 5 Discussion

Leopards in this unprotected, human‐dominated and highly fragmented habitat mosaic rely on a broad, diversified diet including almost three dozen different prey species. This wide‐ranging, inclusive diet is consistent with established leopard feeding ecology which shows the leopard to have the broadest diet of all large predators (Hayward et al. 2006; Kittle et al. 2014). The diversity index results further illustrate the varied, generalist nature of the diet, incorporating numerous prey species with a relatively equitable distribution among them. These results highlight the leopards' adaptability in utilizing available resources in their surroundings, with no species occurring in > 1/5 of scats and no species represented by > ~1/5 of biomass. These results support the generalist predation hypothesis.

Similarly supporting the opportunistic generalist hypothesis, a small species—black‐naped hare (2.5 kg)—was the most frequently consumed prey, but was also the most abundant on the landscape, with selectivity analysis showing consumption in proportion to availability. This result suggests that this common species—which had the highest RAI of all available prey species—represents an important, reliable food source for leopards in this region. However, barking deer may be the most important prey species in the system for leopards as it was the 2nd most frequently detected species in scats, represented ~1/5 of biomass consumed and was moderately preferentially selected. The average weight of barking deer (25.5 kg) falls almost exactly within the size range of most preferred leopard prey globally (23–25 kg; Hayward et al. 2006). In fact, barking deer could be viewed as almost ideal leopard prey as this species fulfills all the preferred characteristics for leopards in that they are the right size, occur in small groups, in dense habitat and provide relatively little threat to the leopard (Hayward et al. 2006). These factors suggest that this species likely represents a key resource in this landscape. The dietary importance of barking deer, coupled with moderate evidence of selection supports the medium‐sized prey hypothesis.

There was also some support for the large prey species hypothesis in that the much larger sambar (adults 163–215 kg; Santiapillai et al. 1981) was also moderately preferred by leopards and provided a considerably greater share of biomass in the diet than its frequency in the diet. The only species with a higher selectivity index than sambar was the domestic cat, but cats were detected with far less frequency and represented a very low proportion of consumed biomass. Selection for sambar may maximize profitability for leopards (Stephens and Krebs 1986) and has been previously observed in Sri Lanka (Kittle, Watson, and Fernando 2017). This selection by leopards for larger, more profitable prey may be expected where dominant intraguild competitors are absent (Stephens and Krebs 1986).

Domestic dogs were represented in < 1/10th of scats and represented a roughly similar proportion of biomass consumed but were moderately avoided by leopards according to their availability on the landscape. The presence of domestic dogs near human settlements or accompanying people on the landscape may heighten the likelihood of confrontation with humans, a risk factor that carnivores often avoid (Ogara et al. 2010). However, this relative avoidance also suggests that there is sufficient wild prey available to leopards, and they are not compelled to prey on domestic species, an observation that supports the available wild prey hypothesis. Although there was some evidence of selection for domestic cats, the proportion of the leopard's prey base that this represents is minimal. Combined with the fact that cats in the tea estate landscape are essentially feral with actual ownership rare, ensures that this is not a significant concern for human–leopard coexistence. Additionally, domestic goats and cattle were not represented in the diet at all. In this region of the Central Highlands, these species are typically not grazed freely but kept in walled pens with fodder brought to them, which reduces the opportunity for them to fall prey to leopards (Uduman et al. 2022). Despite the relatively low importance of domestic species in leopard diet in the study area, which is a positive aspect for long‐term human–leopard coexistence, there remains a perception that leopards represent a serious threat to domestic dogs here (Uduman 2019).

Leopards showed even greater avoidance of wild boar than domestic dogs. This has been observed previously in Sri Lanka (Kittle et al. 2014; Kittle, Watson, and Fernando 2017) and may be due to the threat that wild boar poses to all but the largest of predators (Jędrzejewski et al. 1992; Hayward et al. 2012). This highlights the leopards' capacity to evaluate the costs and hazards linked to prey selection.

Similarly, the reason that porcupines were moderately avoided may have been the threat posed to leopards from their defensive capabilities (Mori et al. 2013). In the Dunumadallawa forest reserve in Sri Lanka's Central Highlands, porcupine was found to be the most common prey species in leopard diet (Kittle et al. 2014), however, this may be due to greater abundance and/or higher relative availability of porcupine in Dunumadallawa, or may simply be the result of individual specialization by a single resident leopard in that 5 km^2^ study area (Araujo et al. 2011; Kittle et al. 2014).

Another key finding from this research was the importance of primate species in leopard diets, as the two primate species—Sri Lanka toque monkey and Sri Lanka purple‐faced leaf monkey—comprised almost ¼ of the diet in terms of both frequency and biomass. This is another pattern that has previously been widely observed, with primates often an important component of leopard diet globally (Hart et al. 1996; Zuberbühler and Jenny 2002; Hayward et al. 2006; Nakazawa 2019; Palei et al. 2021). Primate availability was not possible to estimate from remote camera data due to their arboreal tendencies; however, extensive field observations suggest that these species are not common in the tea estate landscape (Kittle and Watson, personal observation) and therefore may represent a preferred prey here. Alternately, it is likely that primate availability is higher in neighboring forests—certainly with respect to langurs—and they may be preyed upon in these forests with scat subsequently deposited within the tea‐dominated areas.

## 6 Conclusions and Recommendations

This study provides essential insights into the feeding habits of leopards in the UKRB, an unprotected, fragmented and human‐dominated ecosystem. Interestingly, there was support for all three of the hypotheses related to leopard predation patterns, which suggests that leopards employ a nuanced approach to predation. Results support previous observations of the leopard as a generalist, opportunistic predator with a broad diet and a moderate preference for medium‐sized (~23–25 kg) prey (Hayward et al. 2006), while also supporting previous observations in Sri Lanka suggestive of preference for larger prey (Kittle, Watson, and Fernando 2017).

The varied diet observed, lacking strong selection and predominant species, was consistent with a generalist, opportunistic predator and underscores the ecological adaptability of leopards and their capacity to exploit prey of various sizes and types. This flexibility is probably essential for their survival in such landscapes. Awareness of this broad and diverse feeding habit can inform conservation measures, highlighting the need to maintain prey diversity.

However, not all species may be equal, with barking deer likely a key prey resource in this landscape. Ensuring that barking deer habitat requirements continue to be met here might be a particularly useful management enterprise. Additionally, although relatively rare on the landscape, sambar also represent an important resource that provides a disproportionately substantial proportion of the leopard's diet. In a similar landscape in India, it was also shown that these two species were key components of leopard diet (Sidhu et al. 2015). In these regions sambar has been shown to prefer forested, rugged habitats (Kushwaha et al. 2004) whereas barking deer utilize shrub grasslands (Kushwaha et al. 2004; Teng et al. 2004) and plantation landscapes with their mix of tall shrubs, trees and open grass patches (Sidhu et al. 2015). This suggests that the mosaic aspect of this Central Highlands landscape, which offers a variety of habitat types, is important for maintaining a wide and healthy natural prey base for leopards.

Study results clearly indicate that wild prey availability remains sufficient in this region to support the extant leopard population, with the overwhelming proportion of observed leopard diet comprised of wild species. However, despite domestic prey representing a relatively small component (< 15%) of leopard diet here, the predation on dogs in particular is highlighted as a problem by community members (Uduman 2019), raising substantial conservation challenges. Dogs in the tea estate landscape, many of which are owned but unrestricted (Butler et al. 2014), are closely associated with people, often accompanying them into the field during the day and returning with them to communities at night. As has been documented in India, it is from within these isolated communities, typically at night, that leopards appear to hunt them (Daniels 2009; Kittle & Watson, personal communication). A solution to this issue might be to treat dogs at night as cattle and goats are treated on this landscape, and ensure that they are kept indoors, or if they are needed as watchdogs, within strong, secure outdoor pens. As observed by Amerasinghe (1983), sustainable coexistence entails balancing animal conservation and human livelihoods.

A further recommendation is to expand ongoing awareness initiatives targeting tea estate worker communities to: (1) educate people about the ecological significance of leopards as apex predators and their relevance in sustaining biodiversity, and (2) reduce the negative perception of leopards by highlighting the fact that leopards mostly prey on wild species, including those (e.g., rodents, hares, deer) that cause the most damage to cultivation. These programs are required to maintain coexistence between humans and wildlife.

Regarding potential study shortfalls, scat collection for this study was undertaken opportunistically during ongoing remote camera surveys. Nevertheless, the grid design of the camera array ensured that the entire area was comprehensively covered, in which scat samples were opportunistically collected. However, a more systematic approach to scat collection may be useful in the future to ensure that coverage is even and to avoid any possible bias inherent in concentrating search activities. Another shortfall was the absence of abundance, and therefore prey selection, data for primates on the landscape. With such a prominent role in leopard diet it is necessary to determine whether leopards are preferentially targeting these arboreal species, something that could not be done with the employment of ground‐based remote cameras.

In terms of future directions, in addition to quantifying primate availability on the landscape, it would be beneficial to conduct further research to determine the impacts of human activities on leopard behavior, prey availability, and habitat utilization, as understanding how anthropogenic variables influence predator–prey dynamics. This can provide data‐driven insights for conservation strategies.

Overall, this study stresses the relevance of maintaining a natural, diverse prey base, and taking proactive measures to ensure that predation on domestic species, currently at a low level, does not increase. These management directions, together with the ecological flexibility of leopards, can act to promote human–leopard coexistence and the survival of the leopard in unprotected, fragmented landscapes like the UKRB.

## Author Contributions

**P. H. Suranga Chanaka Kumara:** conceptualization (equal), data curation (lead), formal analysis (lead), investigation (equal), methodology (equal), project administration (supporting), software (lead), validation (equal), visualization (lead), writing – original draft (equal). **Andrew M. Kittle:** conceptualization (equal), data curation (supporting), formal analysis (supporting), funding acquisition (equal), investigation (equal), methodology (equal), project administration (supporting), resources (equal), software (supporting), supervision (lead), validation (equal), writing – original draft (equal). **Anjali C. Watson:** conceptualization (equal), data curation (supporting), formal analysis (supporting), funding acquisition (equal), investigation (equal), methodology (equal), project administration (supporting), resources (equal), supervision (supporting), validation (equal), writing – review and editing (equal). **Sandun J. Perera:** conceptualization (equal), funding acquisition (lead), investigation (equal), methodology (equal), project administration (lead), resources (equal), supervision (supporting), writing – review and editing (equal). **Nimalka Sanjeewani:** conceptualization (equal), data curation (supporting), formal analysis (supporting), methodology (supporting), project administration (supporting), software (supporting), supervision (supporting), visualization (supporting). **Saminda P. Fernando:** formal analysis (supporting), methodology (supporting), supervision (supporting).

## Funding

This study was supported by the Sabaragamuwa University Research Grant No. SUSL/RG/2019/02 of the Sabaragamuwa University of Sri Lanka and additional support from CERZA Conservation and the Whitley Fund for Nature.

## Conflicts of Interest

The authors declare no conflicts of interest.

## Supporting information


**Appendix S1:** Camera trap locations.
**Appendix S2:** Model validation for prey importance (biomass) analysis using Lumetsberger et al.'s (2017) non‐linear equation.
**Appendix S3:** Relationship of prey weight with biomass contribution and capture frequency.

**Figure S1:** Residual Plot: Evaluating Model Accuracy for Biomass Estimation.

**Figure S2:** Model Fit: Observed vs. Predicted Biomass for prey species validating the Lumetsberger et al. (2017) non‐linear biomass model.

**Figure S3:** Log–log regression of prey weight vs. biomass contribution.

**Figure S4:** Scatter plot between the prey weight vs. frequency of prey capture.

## Data Availability

Raw data are tabulated within the manuscript and supporting information provided as appendices: camera trap locations (Appendix S1), model validation for prey importance (biomass) analysis using the Lumetsberger et al.'s (2017) nonlinear equation (Appendix S2), and the relationship of prey weight with biomass contribution and capture frequency (Appendix S3).
