# Supplementary material for: Leopards Exhibit Nuanced Predation Patterns but Rely on Wild Prey in a Human‐Dominated Agricultural Landscape in the Central Highlands of Sri Lanka
Source: Ecol Evol. 2026 Feb 11;16(2):e73027. doi: 10.1002/ece3.73027 (PMC12894783; doi:10.1002/ece3.73027)
Supplement: Supplementary file 1 — Appendix S1: Camera trap locations. Appendix S2: Model validation for prey importance (biomass) analysis using Lumetsberger et al.'s (2017) non‐linear equation. Appendix S3: Relationship of prey weight with biomass contribution and capture frequency. [file ECE3-16-e73027-s004.docx]

**Appendix S1: Camera Trap Locations**

Phase one camera trap locations - August to October, 2021

|  | Tea Estate / Location Name | N | E | No. of trap nights |
| --- | --- | --- | --- | --- |
| 1 | Lethanty | 6°52'53.41" | 80°33'42.26" | 85 |
| 2 | Broad Oak | 6°53'28.41" | 80°33'18.52" | 85 |
| 3 | UDK | 6°53'47.54" | 80°32'36.89" | 78 |
| 4 | Velioya G1 | 6°53'47.54" | 80°32'36.89" | 85 |
| 6 | St. Helliers | 6°55'2.24" | 80°32'0.07" | 85 |
| 8 | Eagles_Ridge_Log_Cabin | 6°52'40.30" | 80°34'46.28" | 85 |

Phase two camera trap locations - December 2021 to August 2022

|  | Tea Estate / Location Name | N | E | No. of trap nights |
| --- | --- | --- | --- | --- |
| 1 | Dikoya | 6° 52' 45.45" | 80° 35' 48.70" | 22 |
| 2 | Dikoya | 6° 52' 37.56" | 80° 35' 47.08" | 12 |
| 3 | Batalgala | 6° 52' 54.86" | 80° 36' 54.76" | 248 |
| 4 | Batalgala | 6° 52' 53.19" | 80° 37' 7.85" | 202 |
| 5 | Batalgala | 6° 52' 53.19" | 80° 37' 7.85" | 46 |
| 6 | Fordyce | 6° 52' 11.99" | 80° 37' 40.60" | 87 |
| 7 | Fordyce | 6° 51' 56.67" | 80° 38' 2.12" | 87 |
| 8 | Ingestre | 6° 50' 52.84" | 80° 38' 59.13" | 247 |
| 9 | Ingestre | 6° 50' 35.02" | 80° 39' 30.70" | 201 |
| 10 | Ingestre | 6° 50' 35.02" | 80° 39' 30.70" | 46 |
| 11 | Invery | 6° 53' 9.28" | 80° 38' 5.35" | 207 |
| 12 | Robgill | 6° 49' 45.62" | 80° 39' 48.06" | 74 |
| 13 | Robgill | 6° 49' 47.68" | 80° 41' 2.90" | 157 |

WWCT camera trap locations - August to December 2016

|  | Tea Estate / Location Name | N | E | No. of trap nights |
| --- | --- | --- | --- | --- |
| 14 | Dunkeld | 6°51'19.98" | 80°34'12.61" | 29 |
| 15 | Kelenaya | 6°51'14.51" | 80°33'46.07" | 32 |
| 16 | Kew | 6°47'42.22" | 80°38'22.95" | 34 |
| 17 | Kew | 6°48'16.9" | 80°37'58.13" | 34 |
| 18 | Kew | 6°48'4.68" | 80°38'7.32" | 16 |
| 19 | Venture | 6°48'30.34" | 80°37'26.33" | 33 |
| 20 | Norwood | 6°50'3.55" | 80°36'6.98" | 33 |
| 21 | Alton | 6°47'23.46" | 80°38'10.86" | 29 |
| 22 | Norwood | 6°49'23.3" | 80°36'35.89" | 31 |
| 23 | Fairlawn | 6°45'51.38" | 80°37'40.73" | 30 |
| 24 | Gouravilla | 6°48'13.21" | 80°36'27.72" | 28 |
| 25 | Glentilt | 6°50'16.94" | 80°34'56.84" | 30 |
| 26 | Gouravilla | 6°47'24.29" | 80°36'48.82" | 32 |
| 27 | Strathspey | 6°46'4.4" | 80°36'17.35" | 33 |
| 28 | Strathspey | 6°46'19.13" | 80°35'11.22" | 33 |
| 29 | Strathspey | 6°46'46.02" | 80°35'6.76" | 33 |
| 30 | Mocha | 6°47'48.77" | 80°34'39.14" | 33 |
| 31 | Brunswick | 6°49'21.5" | 80°34'50.48" | 32 |
| 32 | Mocha | 6°48'4.57" | 80°33'47.89" | 32 |
| 33 | Gartmore | 6°46'44.22" | 80°33'34.2" | 32 |
| 34 | Gartmore | 6°46'40.22" | 80°33'30.38" | 32 |
| 35 | Gartmore | 6°47'43.22" | 80°32'48.95" | 32 |
| 36 | Glenugie | 6°47'24.97" | 80°35'57.48" | 32 |
| 37 | Glenugie | 6°47'32.17" | 80°36'3.71" | 32 |
| 38 | Brunswick | 6°49'21.61" | 80°35'38.11" | 30 |
| 39 | Dunkeld | 6°51'24.95" | 80°34'3.94" | 12 |
| 40 | Osborne | 6°52'16.38" | 80°33'16.2" | 35 |
| 41 | Osborne | 6°53'7.15" | 80°32'34.98" | 35 |
| 42 | Osborne | 6°53'18.09" | 80°32'32.1" | 35 |
| 43 | Moray | 6°48'30.02" | 80°31'41.38" | 35 |
| 44 | Peak Wilderness | 6°49'24.75" | 80°30'38.2" | 35 |
| 45 | Laxapana | 6°50'29.0" | 80°31'8.26" | 35 |
| 46 | Laxapana | 6°50'19.71" | 80°31'26.9" | 35 |
| 47 | Hapugastenna | 6°52'34.86" | 80°31'48.72" | 33 |
| 48 | Hapugastenna | 6°52'50.16" | 80°31'24.02" | 33 |
| 49 | Laxapana | 6°50'30.62" | 80°32'14.24" | 34 |
| 50 | Forres | 6°49'2.21" | 80°32'37.64" | 34 |
| 51 | Moussekelle | 6°51'43.45" | 80°32'52.38" | 32 |
| 52 | Glentilt | 6°50'41.15" | 80°34'26.43" | 32 |
| 53 | Glenugie | 6°48'24.76" | 80°35'45.42" | 23 |

**Appendix S2: Model Validation for Prey Importance (Biomass) Analysis using Lumetsberger et al.’s (2017) non-linear equation**

**Residual Plot: Evaluating Model Accuracy for Biomass Estimation**

The residual plot in Figure S1 below depicts the discrepancies between observed and anticipated biomass, indicating that the model correctly estimates biomass for smaller prey but underestimates contributions from larger prey such as barking deer and sambar, hence implying the necessity for model refining.

*Figure S1. Residual Plot: Evaluating Model Accuracy for Biomass Estimation*

**Validation plots of the Lumetsberger et al. (2017) non-linear biomass model.**

The Figure S2, comparing observed and predicted biomass reveals a strong correlation between expected and actual values for most prey species validating the Lumetsberger et al. (2017) non-linear biomass model.

*Figure S2. Model Fit: Observed vs Predicted Biomass for prey species validating the Lumetsberger et al. (2017) non-linear biomass model*

**Appendix S3: Relationship of prey weight with biomass contribution and capture frequency**

The log-log regression (Figure S3) demonstrates the correlation between prey weight and estimated biomass contribution, revealing a positive correlation where larger prey typically provide more biomass, with certain exceptions (e.g., black-naped hare and sambar) reflecting species-specific dietary habits.

*Figure S3. Log-log regression of prey weight vs biomass contribution.*

The scatter plot (Figure S4) shows that smaller prey, such as the black-naped hare and Sri Lanka toque monkey, are captured more frequently, while bigger prey like sambar is consumed less often, but represents a significant portion of the leopard's diet owing to its greater biomass contribution.

*Figure S4. Scatter plot between the prey weight vs. frequency of prey capture.*
