# Supplementary figures and images for: Leopards Exhibit Nuanced Predation Patterns but Rely on Wild Prey in a Human‐Dominated Agricultural Landscape in the Central Highlands of Sri Lanka
Source: Ecol Evol. 2026 Feb 11;16(2):e73027. doi: 10.1002/ece3.73027 (PMC12894783; doi:10.1002/ece3.73027)

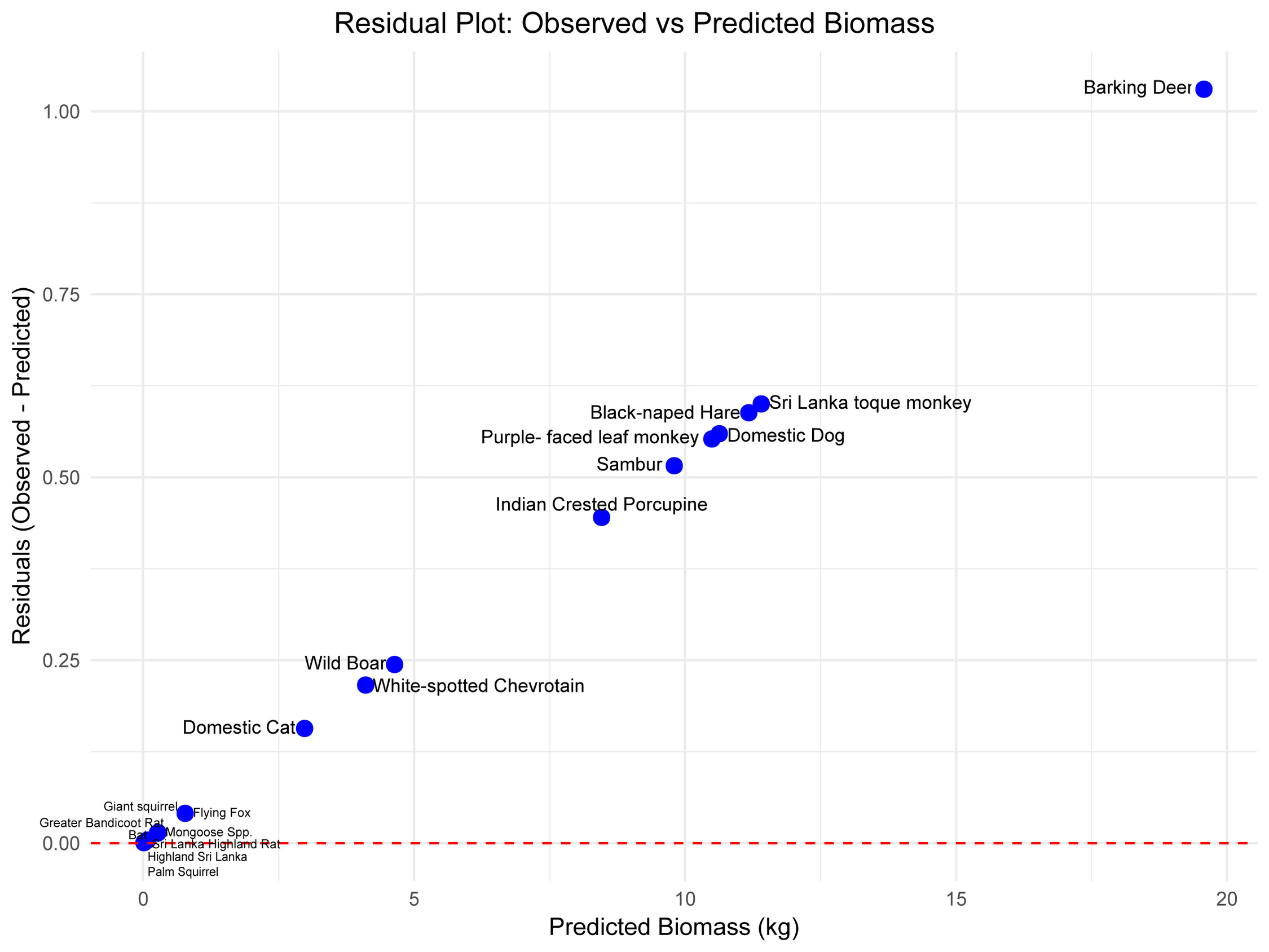

Supplement: Supplementary file 2 — Figure S1: Residual Plot: Evaluating Model Accuracy for Biomass Estimation. [file ECE3-16-e73027-s003.tif]

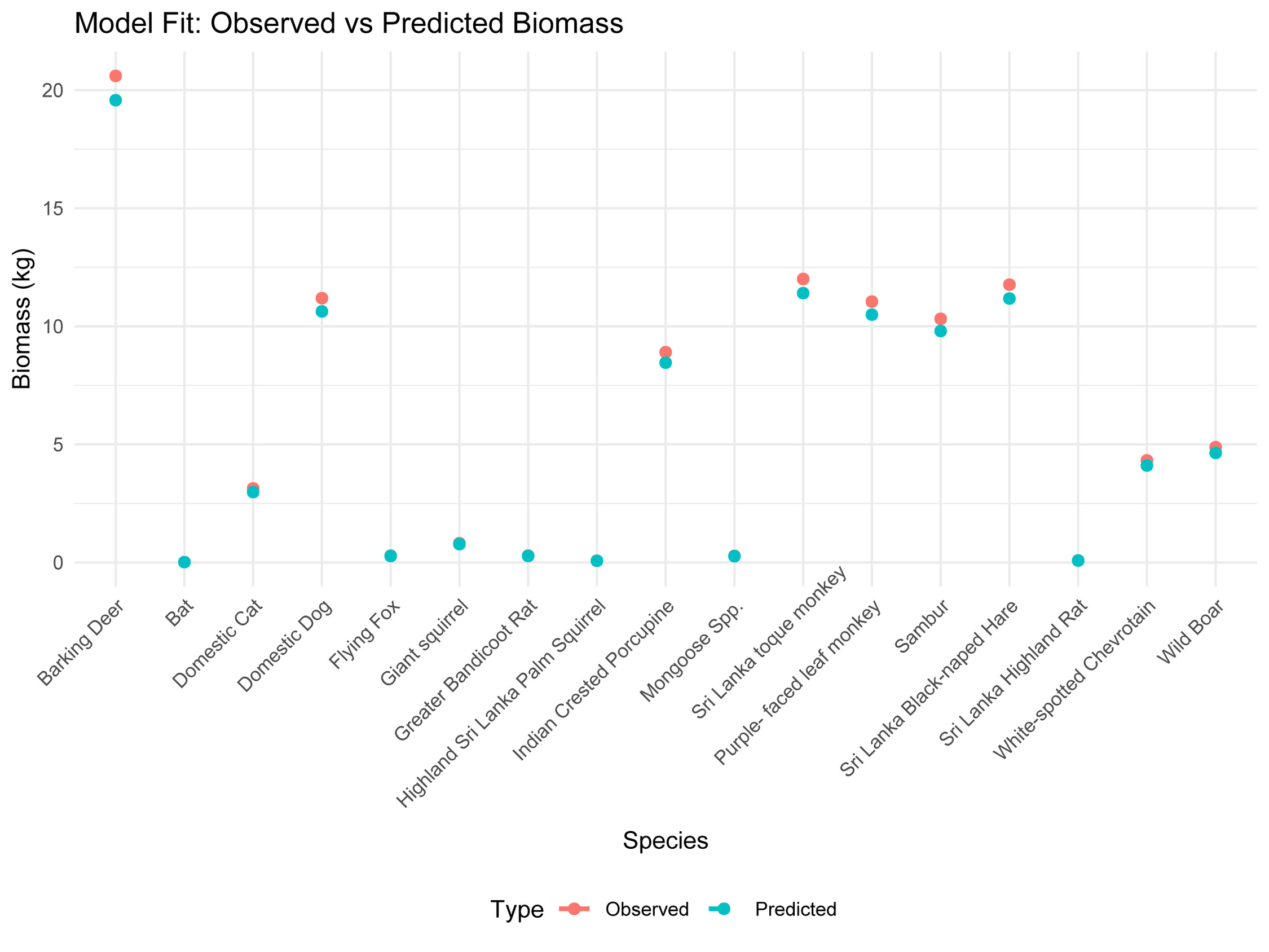

Supplement: Supplementary file 3 — Figure S2: Model Fit: Observed vs. Predicted Biomass for prey species validating the Lumetsberger et al. (2017) non‐linear biomass model. [file ECE3-16-e73027-s001.tif]

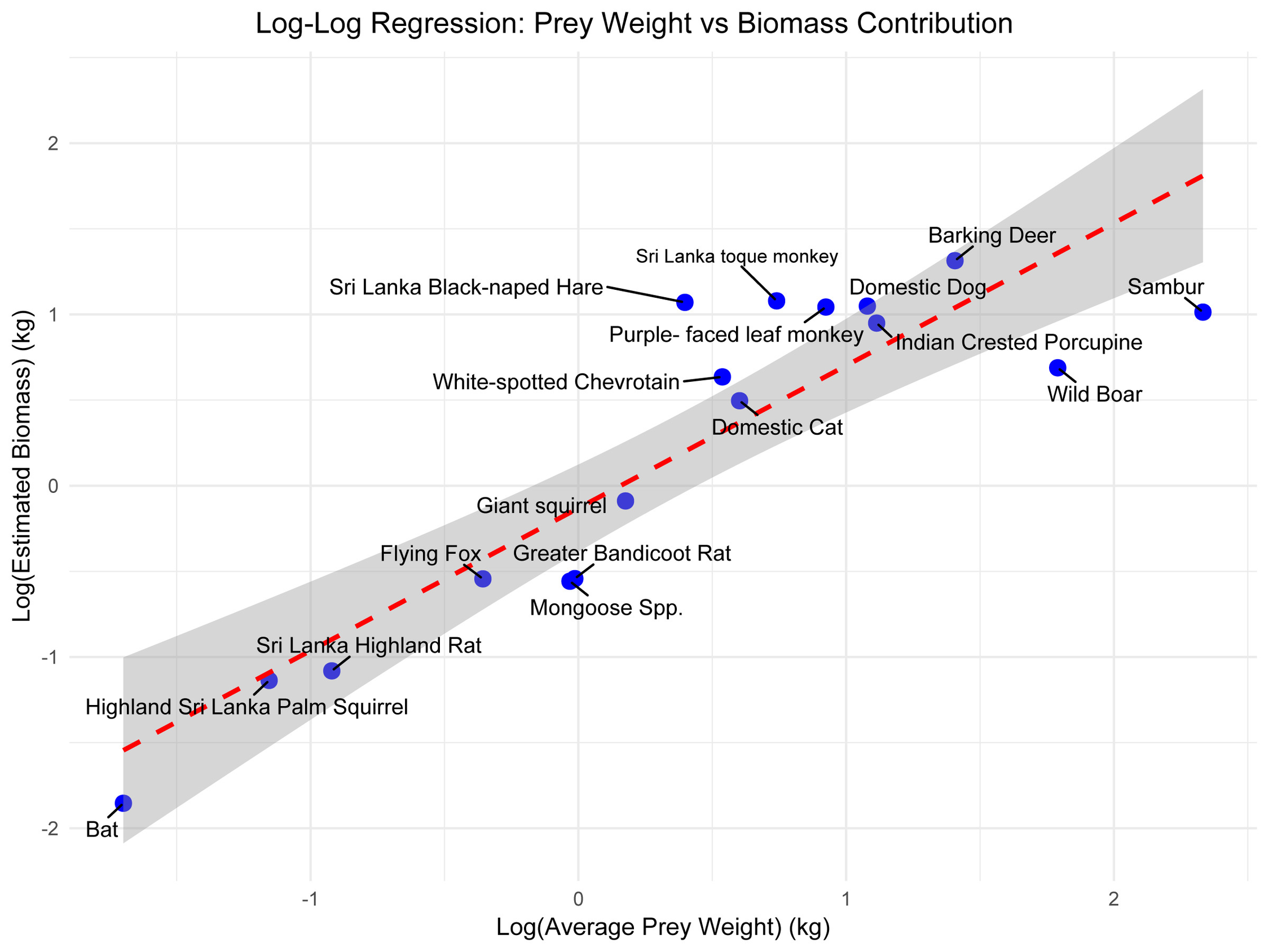

Supplement: Supplementary file 4 — Figure S3: Log–log regression of prey weight vs. biomass contribution. [file ECE3-16-e73027-s002.tif]

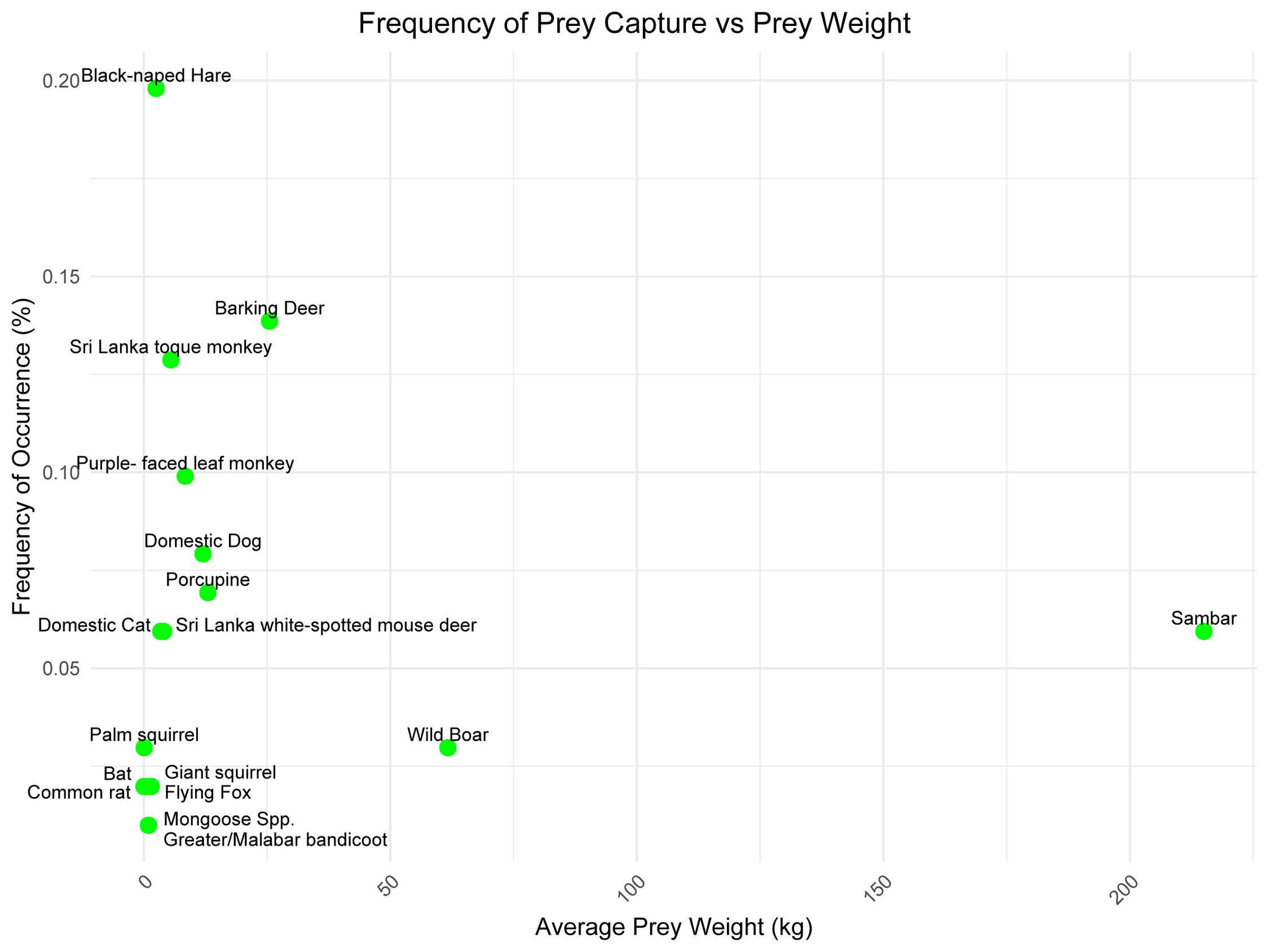

Supplement: Supplementary file 5 — Figure S4: Scatter plot between the prey weight vs. frequency of prey capture. [file ECE3-16-e73027-s005.tif]
